# The recent advancement of outdoor performance of perovskite photovoltaic cells technology

**DOI:** 10.1016/j.heliyon.2024.e36710

**Published:** 2024-08-22

**Authors:** Getnet M. Meheretu, Ababay Ketema Worku, Moges T. Yihunie, Richard K. Koech, Getasew A. Wubetu

**Affiliations:** a Bahir Dar Energy Center, Bahir Dar Institute of Technology, Bahir Dar University, Ethiopia; b Department of Physics, College of Science, Bahir Dar University, Ethiopia; c Department of Mathematics, Physics and Computing, School of Science and Aerospace Studies, Moi University, Kenya; d Department of Physics, College of Natural and Computational Sciences, Addis Ababa University, Ethiopia

**Keywords:** Perovskite solar cells, Irradiance, Outdoor testing, Performance, Long term stability

## Abstract

Perovskite solar cells achieved a record for power conversion efficiency of over 26 % for single junction cells and 34 % for planar silicon/perovskite tandems. These cells can be manufactured from low-cost materials with low-tech production techniques. As a result, it attracted great attention for future solar technology and multiple performance and stability studies have been reported in research articles. This work summarizes recent (2019–2023) reports on outdoor performance and stability tests of perovskite solar cells and modules in different locations and climate conditions. The review realized that there are limited works on outdoor testing of perovskite solar cells, and found only one year is the maximum long-term outdoor stability reported as at the time this review was conducted. The reports of this review demonstrated that accelerated aging tests of perovskite solar cells under harsh conditions such as elevated temperature, damp heat, and high relative humidity cannot replace realistic outdoor testing. As a result, studying the performance and stability of perovskite solar cells and modules under real outdoor conditions is very important to realize its commercialization.

## 1 Introduction

In the dynamic landscape of renewable energy, perovskite solar cells (PSCs) have emerged as promising contenders due to their remarkable advancements in power conversion efficiency (PCE) over a short span of research and development. However, the journey towards their widespread commercialization faces a crucial bottleneck: the transition from controlled laboratory conditions to real-world outdoor environments. Traditional testing protocols, confined within laboratory walls, fail to capture the nuances of outdoor exposure, where fluctuating weather patterns and varying peak sun hours significantly influence performance and stability. Indeed, outdoor assessments represent a pivotal but largely uncharted territory in the quest to commercialize perovskite PV modules. The in adequate literature available on this subject underscores a glaring research gap that warrants urgent attention. Despite the pivotal role of outdoor testing in gauging long-term stability and performance, fewer than 30 published reports exist on the outdoor behavior of perovskite solar cells to date [1].

Existing outdoor characterizations of PSCs often overlook the crucial interplay between solar cell parameters such as short-circuit current density (J_SC_), open circuit voltage (V_OC_), and fill factor (FF) and the dynamic outdoor conditions, such as irradiance and temperature fluctuations PSCs [1]. Consequently, a pressing need arises for comprehensive research to bridge this gap and deepen our understanding of perovskite device behavior in authentic outdoor settings. Researchers have responded to this challenge by innovating various surface and interface engineering techniques aimed at bolstering the outdoor performance of perovskite devices. However, accessing extensive outdoor testing data remains critical for refining degradation models and elucidating acceleration factors essential for projecting the lifetime of PSCs accurately. While accelerated aging tests offer insights into device long term stability, their reliability hinges on complementing them with robust outdoor testing protocols. Timely outdoor assessments, particularly for large-scale devices, become imperative to ensure the viability of perovskite technology in real-world applications [2].

Even though perovskite solar cells (PSCs) seem promising for outdoor use, there haven't been many high-quality studies on how well they actually perform outside. This means we need to do more research to understand how they work in real-world conditions. Encapsulated devices dominate outdoor testing reports, leaving non-encapsulated counterparts relatively understudied. Moreover, while rigid devices have garnered attention, flexible counterparts remain largely unexplored in outdoor settings.

In light of these challenges and opportunities, this study endeavors to delve into the outdoor performance and stability of perovskite solar cells, shedding light on their behavior under real-world conditions and charting a course towards their successful commercialization.

**Fig. 1:**
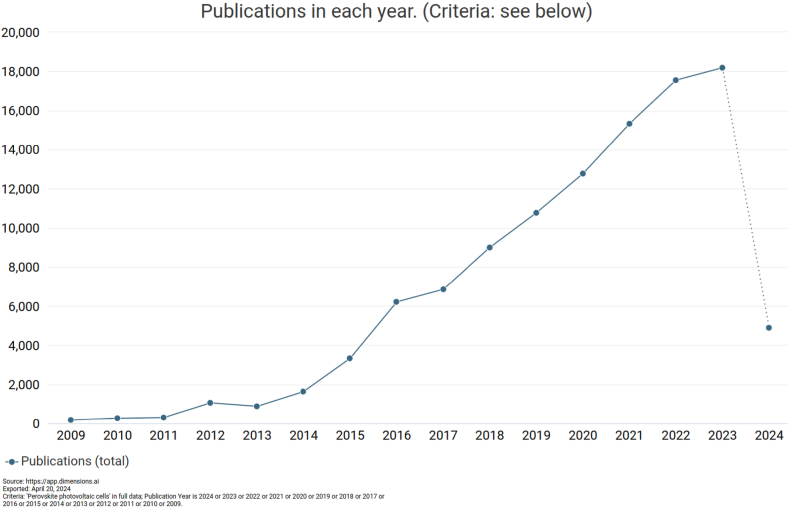
Number of publications searched by the word (Perovskite photovoltaic cells) in their titles. The data were collected from Dimensions research database on April 20, 2024.

As depicted in Fig. 1, there has been a dramatic increase in the number of yearly published research articles focusing on Perovskite photovoltaic cells in recent years. Access to extensive outdoor testing data for perovskite devices is crucial for developing comprehensive degradation models and understanding acceleration factors. Perovskite solar cells (PSCs) that can withstand degradation effects demonstrate stable performance during long-term outdoor operation. While stability tests conducted in the laboratory are typically carried out under constant illumination, outdoor conditions involve continuously varying illumination, leading to distinct testing conditions [3].

## 2 Device architectures

The fundamental elements of a Perovskite Solar Cell (PSC) consist of an anode, a cathode, a Hole Transport Layer (HTL), an Electron Transport Layer (ETL), and a perovskite absorber layer. PSCs are generally categorized into three primary types: traditional Electron-Absorber-Hole (n-i-p), inverted Hole-Absorber-Electron (p-i-n), and, in specific instances, mesoporous structures. In the production process of the n-i-p architecture, the ETL is deposited first, while in the p-i-n architecture, the HTL is initially deposited. The intrinsic perovskite absorber layer usually sandwiched between the HTL and the ETL in a PSC [4].

**Fig. 2:**
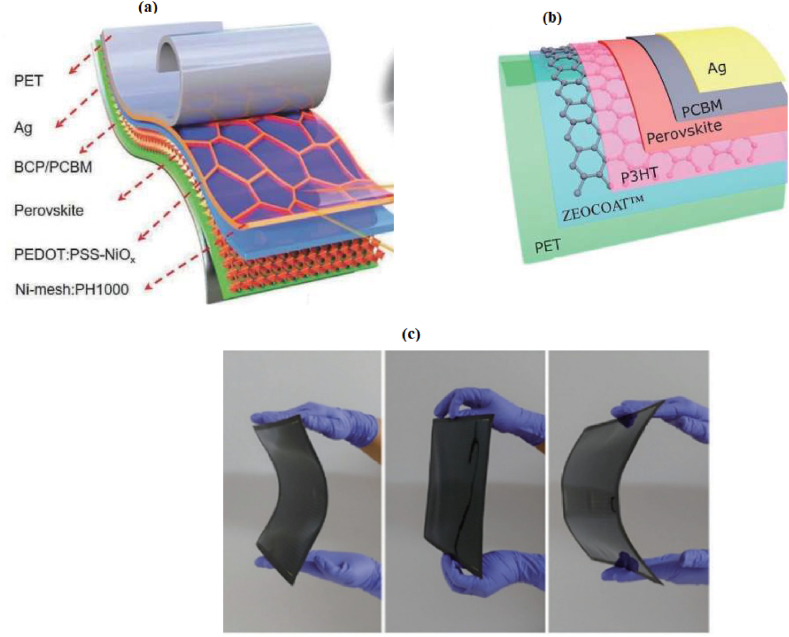
**a**) The flexible perovskite solar cells' (FPSC) structure. *Reprinted from* [5] *under creative common (CC-BY)license Copy right 2020, John Wiely and Sons.***b)** Schematic diagram of a particular FPSC design architecture**.***Reprinted from* [6] *Copyright 2016, with permission from Elsevier***c)** Photographic image of FPSC.*Reprinted from* [7] *copyright 2020, Royal Society of Chemistry.*

**Fig. 3:**
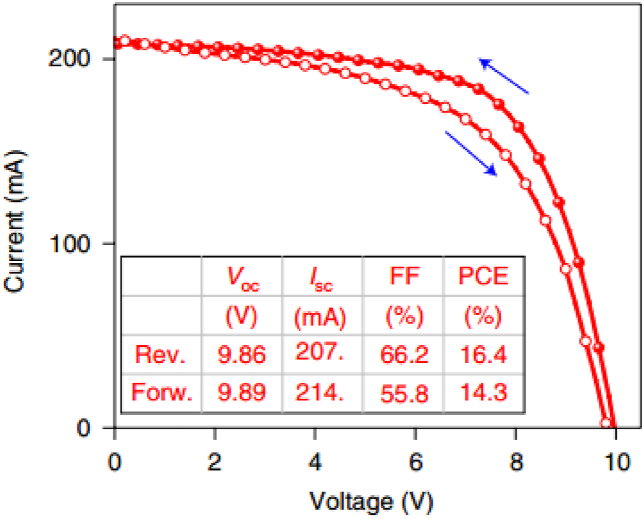
*Current density-Voltage (J-V) characteristics of a perovskite solar module under 1Sun outdoor illumination for both forward and reverse scan as indicated by the arrows*. Reproduced with permission from Ref. [8] copyright 2022, Springer Nature.

In a PSC device, electrons generated in the perovskite absorber are transported to the ETL, which resides at the conductive band of the perovskite layer, while holes are transported to the HTL. Both the ETL and HTL facilitate the movement of electrons and holes towards the respective electrodes and prevent their reverse flow. A comprehensive assessment of the ETL, perovskite absorber, HTL, and their interfaces is crucial for the successful fabrication of high-performance PSCs. During the fabrication process of PSCs, the choice of materials and the quality of interfaces play pivotal roles. As depicted in Fig. 2c, a typical perovskite device comprises an electrode, a conducting substrate, an absorber layer, and charge carriers [4] (see Fig. 3).

The outdoor stability of perovskite device depends on the device architecture, in particular the hole transport layer (HTL) [3,8]. Many efforts have been applied for fabrication of large scale perovskite solar module (PSM), for real operation. Most PSCs whose outdoor stability reported in literature has conventional (n-i-p) device architectures, while outdoor stability tests of inverted (p-i-n) PSCs are not adequately reported as compared to the former one [9]. Thus, it is very essential to undertake comprehensive study of outdoor stability tests to understand the performance evolution of PSCs of different device architectures, mesoporous or planar, conventional (n-i-p) or inverted (p-i-n) as each device architecture may have different degradation patterns [3]. Pescetelli et al. [2] manufactured large scale graphene perovskite panels of area 0.5 m^2^ by connecting 40 modules. They demonstrated perovskite panel as large as 4.5 m^2^ and by integrating these panels they produce a peak power of more than 250W, evidenced that the technology can be scalable. Pescetelli's team [8] also developed graphene and other two-dimensional materials for interface engineering of perovskite devices. Pescetelli's group developed large number of modules, opted for solution inspired by silicon technology with optimal trade-off between the PCE and reproducibility [8].

Xu et al. [10] studied the two weeks outdoor stability of perovskite tandem devices and proposed the possible mechanism to improve its stability. The group used highly transparent interconnecting layers (ICL) using Atomic Layer Deposition (ALD) on SnO_2_ films and ultrathin layer of PEDOT: PSS to investigate the effect of metal free ICL on the double cation perovskite that has a composition of FA_0.8_Cs_0.2_ Pb(Br_0.4_I_0.6_)_3_ to study the performance and outdoor stability of the perovskite tandem device. Wide band gap perovskite (E_g_ = 1.78 eV) used as the front cell. Pitchaiya et al. [11] used state of the art techniques for fabrication of large-area, HTM free C-PSC technology with an active area of about 88 cm^2^ for the first time with the device configuration: FTO/c-TiO_2_/mp-TiO_2_/perovskite (CH_3_NH_3_PbI_3-x_Cl_x_)/C [11].

Bastiani et al. [8,12] studied the outdoor performance of Monolithic Perovskite/Silicon Tandem Photovoltaics in a hot and humid climate for six-moths (December to June) in Saudi Arabia. The team placed three identical bifacial perovskite/silicon tandem solar cells of p-i-n architecture with south facing test-field at an angle of 25^0^ with latitude. Babics et al. studied the outdoor performance of Perovskite/silicon tandem solar cells over one year in the Red Sea coast of Saudi Arabia where the environment is hot and humid. Babics and his colleagues fabricated perovskite/silicon tandem encapsulated devices using Cs_0.05_ MA_0.14_ FA_0.81_Pb(Br_0.72_I_0.28_)_3_ which has a band gap of 1.68 eV and passivated with Ni O_x_ which acts as a hole transport layer (HTL) [13].

Emery et al. [14] prepared p-i-n (inverted) device with configuration ITO|2PACz|perovskite|C_60_|SnO_2_|Cu and peroskite composition Cs_0.15_FA_0.85_PbI_2.55_Br_0.45_. Jošt et al. [15] registered the useful weather data and tracked the output power at the maximum power point (MPP). For this outdoor study, they used inverted (p-i-n) device with architecture glass|ITO|MeO-2PACz |perovskite|C_60_|SnO_2_|Cu. The perovskite layer they used has a mixed triple cation with composition of Cs_0.05_(FA_0.83_MA_0.17_)Pb_1.1_(I_0.83_Br_0.17_)_3_. Gao et al. [12] stated that monolithic perovskite tandem solar cells configuration shall be optimized based on the band gap energy (E_g_), temperature, and thickness under field testing real outdoor conditions. They fabricated perovskite tandem configuration glass/ITO/PTAA/wide-E_g_ perovskite/C60/ALD-SnO_2_/Au/PEDOT:PSS/narrow-E_g_ perovskite/C_60_/BCP/Cu where the wide E_g_ (1.76 eV) has Cs_0.2_FA_0.8_PbI_1.86_Br_1.14_ and the narrow E_g_ (1.22 eV) has a composition of MA_0.3_FA_0.7_Pb_0.5_Sn_0.5_I_3_. The printable device for this testing contains TiO_2_/ZrO_2_/carbon triple layer and prepared on fluorine doped tin oxide (FTO) glass substrates using screen-printing methods [2]. The report presented the outdoor stability of interface engineered infiltered (IND) and layer-to-layer-deposited (BLD) perovskite devices and demonstrated that such devices maintain 52.9 % of its initial PCE after 10 days of performance study. However, STD devices which do not have interface layer drop its initial performance down to 22 % after 10 days of stability assessment.

## 3 Outdoor testing conditions

There are three international summit on organic photovoltaic stability (ISOS) testing protocols for outdoor testing.

(i). ISOS-I involves keeping the device under open circuit (OC) or maximum power point (MPP) at fixed operating voltage. This testing protocol requires only adjusting the outdoor conditions rig.
(ii). ISOS-O-2 protocol involves testing of devices under OC or MPP, with I-V curves measured under natural sunlight.
(iii). ISOS-O-3 involves in situ MPP tracking, as well as measurement of I-V curves under both solar simulator and natural sunlight. This is the most challenging testing protocol under outdoor conditions, perovskite device exposed to solar radiation, temperature, and alternating cycles of illumination and darkness [16].

In order to conduct outdoor testing, the samples shall be mounted at fixed inclination. There are also outdoor testing reports using tracking. For instance, the power generated by bifacial perovskite–silicon tandem cells can increase by 55 % using power trackers instead of fixed mounting [3]. In most outdoor testing, solar cells are maintained near the maximum power point (MPP) than being in open circuit conditions [17]. There are procedures to conduct outdoor performance of PV modules, which can have two sections; instantaneous and long term performance measurement of PV modules under outdoor conditions. Continuous monitoring the PV module performance and weather parameters are required for long term outdoor performance testing [18]. The outdoor performance study has been made for different ranges of temperature, humidity, and irradiance and ISOS-1 (open circuit or MPP tracking) and ISOS-2 testing protocols. For temperature ranges from −10 to 35°C, under outdoor testing, the surface temperature of the PV module can reach as high as 70°C [9]. The most important testing for outdoor operation of perovskite device is testing in combined stressors in very harsh environment. The continuous variations in temperature, humidity, solar radiation intensity and spectrum, makes stability test under outdoor condition very challenging. The outcomes of the existing accelerated aging protocols are not reliable for prediction of outdoor operation lifetime of the devices [3]. There are reports on the performance and stability of perovskite solar cells under standard testing conditions (STC) and outdoor field testing conditions (FTC).

Pescetelli et al. [8] considered all the relevant meteorological data such as solar irradiance, humidity, and temperature (both ambient and panel) recorded in real-time data acquisition system for the outdoor monitoring of their study. Liu et al. [19] studied the outdoor performance of encapsulated perovskite/silicon solar cells in a hot and sunny environment. Aydin et al. [10,20] conducted outdoor measurement on a rooftop in a hot and sunny climate for a monolithic perovskite/silicon tandem solar cells that has a PCE of 25 % to study the dependence of the band gap on temperature. The group used open–rack test configuration on a rooftop for outdoor testing and found that the maximum temperature of the cell under investigation to be 60 °C. As a result, 75 °C is assumed to be the realistic temperature for rooftop measurements at high solar irradiance.

Outdoor stability tests conducted for about 800 h by Xu et al. [10] to show the potential of perovskite tandem devices with metal free ICL [10]. The group used reverse JV-scan at a scan rate of 50 mV/s where the tandem device was held at the maximum power point between the JV-scans, while the single junction device was held at open circuit voltage in between the measurements to study the outdoor operation of such devices. Carlo et al. [21] reported the outdoor photovoltaic parameters of a solar cell under real tropical weather conditions. The group studied two perovskite modules that have an active area of 17 and 50 cm^2^. Using silicon as a reference, the technology was evaluated for 500 h correlating the in-situ I-V measurements with atmospheric variables measured every minute during the light hours. The device outdoor performance and efficiency was obtained using IEC 61853-1 standards. The demonstration shows that the short circuit current density and the power delivered vary linearly with the atmospheric parameters. Outdoor testing has been carried out for PSCs with an active area of 0.09 cm^2^ under the condition that the devices were exposed to natural sunlight during the day and stored in a glove box during the night. JV-scan measurement was then taken twice per day at standard testing conditions. Testing using ISOS-O-1 protocol has been made to estimate the performance of 0.16 cm^2^ solar cells and 4 cm^2^ mini-modules. In this testing condition, JV-scan measurements were conducted three times per day under simulated light during two weeks of outdoor exposure and storing the devices in dark in a glove box during the night. Comparison of the V_OC_ between indoor and outdoor measurement was made at high temperature for sealed perovskite devices of area 0.283 cm^2^ [22]. Pitchaiya et al. [11] studied the performance of large scale PSCs under continuous outdoor exposure in Bergen, Norway climate condition on July 2021. According to this report the champion BLD devices showed significant PV performance when tested even under very harsh environmental conditions such as normal drinking water, cold water, and hot water [11]. The outdoor performance of PSCs varies with location due to the variation in temperature and irradiance. Therefore, pilot performance tests at different locations is every essential [3,8]. Babics group installed a testing rig at fixed tracking positioning towards south tilt angle of 25° and conducted outdoor monitoring throughout a year (April 2021 to April 2022) in a harshest conditions where there is elevated temperature, high solar radiation reaching to 2000 kWh/m^2^ per year, and relative humidity (RH) between 60 % and 90 % during the year [13]. Liu et al. [23] demonstrated a model for perovskite-Si tandem efficiency with respect to spectral composition, intensity level, and temperature and analysed the operating conditions in each climate zone using NASA satellite data. The main parameters investigated are irradiance level, temperature, and humidity [23]. Pitchaiya and his colleagues was reported based on the long term efficiency of carbon-based large-scale perovskite solar cells (C-LSPSCs) under different testing conditions such as 1Sun and 0.1 Sun continuous illumination at ambient conditions, submerged and soaked in water at different temperatures and PH values. According to this report, stability testing conditions working of a small fan powered with C-LSPSC devices stimulates interest in the area [11]. Wright et al. [24] explained the rigorous nature of outdoor testing considering outdoor characterization at King Abdullah University of Science and Technology (KAUST).

## 4 Testing encapsulated devices

Perovskite devices can be tested under outdoor environment with encapsulation. However, most encapsulation has a significant problem to satisfy harsh testing conditions such as elevated temperature, damp heat, and outdoor testing [25], as a result perovskite solar cell outdoor testing reports are very limited [1]. Although majority of the outdoor testing of perovskite solar cells are on encapsulated devices, there are outdoor testing reports on non-encapsulated devices. The outdoor tests of non-encapsulated planar devices fail faster than its mesoscopic counter parts; yet, non-encapsulated mesoscopic devices has short lifetime. Jošt et al. [15] prepared 1 cm^2^ size perovskite devices with an average lab PCE of 18.5 % and placed on the rooftop after encapsulated. Emery et al. [14] demonstrated that using encapsulation it can be possible to retain the initial performance of PSCs for 3 months continuous outdoor illumination.

Encapsulation enables perovskite devices to retain 80 % of its initial PCE after 100h of operation at a temperature of 85 °C and relative humidity of 85 % and 95 % of its initial PCE after 6 h operation at −15 °C [26]. Long term stability of PSCs under outdoor conditions can be ensured only with effective encapsulation in both front and back contacts of the device [26]. Tracking the outdoor stability obviously requires the use of series resistance with the encapsulated perovskite devices. Polymer/Al encapsulated devices maintain about 90 % of its initial efficiency after 500 h whereas polymer/glass encapsulated devices drops more than 25 % under outdoor illumination where the average temperature and relative humidity are 18°C and 38 % respectively. Using 2 mm of Al sheet beside PMMA/SB protective layer enhances the stability of perovskite devices and enables to maintain 90 % of its initial PCE after 500 h of outdoor exposure [26]. It is reported that encapsulation with transparent plastic frame together with an edge seal improve the long term outdoor stability of C-LSPSC device even under very harsh testing conditions [11].

## 5 Testing passivated and non-passivated devices

Paraskeva et al. reported the outdoor performances of two perovskite active layers one with formamidinium chloride (FACl) additive, while the other without any additive. In this outdoor testing, it is reported that the perovskite modules with additives degrade faster than those without additives, implies that the benefit of the additives is not observed. They studied the degradation rates, and observed that the one with additive demonstrated faster degradation under open circuit loading conditions between JV-scans. The diurnal cycle outdoor testing demonstrates that the modules efficiency is high in the morning and low in the afternoon [27]. Liu et al. [19] demonstrated that carbazole additive can reduce non recombination loss and suppress phase segregation under environmental exposure. Liu's group assess the stability of the device with the presence of carbazole additive, and encapsulated device tests has been conducted under outdoor and light soaking conditions at temperature of 85^0^C and RH 85 % and dump heat test. The performance evolution of pristine device and carbazole-treated device has been studied. The carbazole-treated device shows the better stability in comparison with the pristine device. According to the report then carbazole additive can improve the stability of perovskite devices. The presence of the additive enables the device to retain 93 % of its initial performance for more than 43 days in a harsh environment (hot, humid, and damp heat) test. This shows that treatment with carbazole enhance the stability significantly.

Chen et al. [22] investigated a method to improve the performance and stability of perovskite solar cells (PSCs). They focused on a specific device architecture ITO/PTAA/Perovskite/PCBM/BCP/Cu where ITO=Indium Tin Oxide, PTAA = Poly(triarylamine), PCBM = 6,6-Phenyl-C_61_-butyric acid methyl ester, BCP = Bathocuproine, SnO_2_= Tin(IV) oxide, Cu = Copper, and introduced an atomic layer deposition (ALD) processed SnO_2_ layer as a buffer layer between the perovskite and electron transport layer (PCBM). This modification significantly increase the PCE from about 17 % to 20 % and enhanced stability. Devices with the SnO_2_ layer retained over 90 % of their initial efficiency after 600 h in ambient conditions (20–40 % relative humidity) without encapsulation, while control devices without SnO_2_ retains only 70 % efficiency of its initial PCE. The ALD-processed SnO_2_ layer, optimized at a thickness of 30 nm, was key to this improvement of the champion device. This approach demonstrates the potential of incorporating ALD- SnO_2_ layers for significantly improved stability in PSCs, paving the way for future commercialization.

Tian et al. [28] introduced a novel "crystal redissolution" (CR) strategy that allows film formation in ambient air with high humidity, eliminating the need for specialized setups. They incorporated 4-N,N-dimethylamino-4ʹ-Nʹ-methyl-stilbazolium tosylate (DAST) as a key passivating additive. This molecule interacts with CsPbI_3_ to promote the formation of the desired black phase, enhancing device performance, and to passivate the black phase, preventing degradation to the less efficient yellow phase and thereby improving stability. XRD tests confirmed that CR-treated films with DAST remained stable for at least a month in regular air, showcasing the effectiveness of this approach. This method has the potential to significantly simplify production and improve the stability of perovskite solar cells.

## 6 Outdoor performances of perovskite devices

Outdoor performance reports on perovskite solar cells are limited. However, there are some reports conducted by different researchers. Bastiani et al. [29] reported the certified PCE of bifacial tandem exceeds 25 % under outdoor conditions at AM 1.5G and illumination intensity 26 mW/cm^2^. The report made a comparison study of outdoor tests for monofacial and bifacial perovskite/silicon tandems at different band gap energies.

Pescetelli ‘s group [8] developed graphene and other two-dimensional materials for interface engineering of perovskite devices and demonstrated that a maximum PCE of 16.4 % for efficient perovskite solar modules under outdoor characterizations that has been conducted with solar trackers at irradiance of 1000 W/m^2^. The study reported that panels that have an active area of 0.32 m^2^ can give rise to an average power of about 30 W, and average PCE of 9.2 %. The outdoor monitoring of their study, considered all the relevant meteorological data such as solar irradiance, humidity, and temperature (both ambient and panel) recorded in real-time data acquisition system. The PV parameters such as PCE, FF, V_OC_, and J_SC_ are sensitive to temperature and irradiance, which can be controlled by the balance between charge generation and recombination. Pescetelli's group developed large number of modules, opted for solution inspired by silicon technology with optimal trade-off between the PCE and reproducibility [8].

**Fig. 4:**
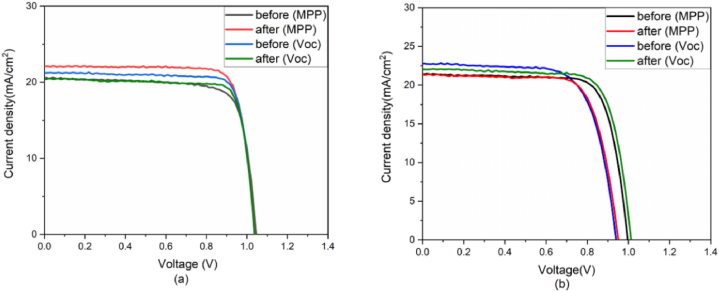
Current density-voltage curves of perovskites cells with additive (a) for type B modules and (b) for type A modules before and after MPP and open-circuit loading for several minutes. Reprinted from Ref. [27] in accordance with Creative Commons Attribution (CC-BY) license 4.0 Copyright 2023,MDPI.

**Fig. 5:**
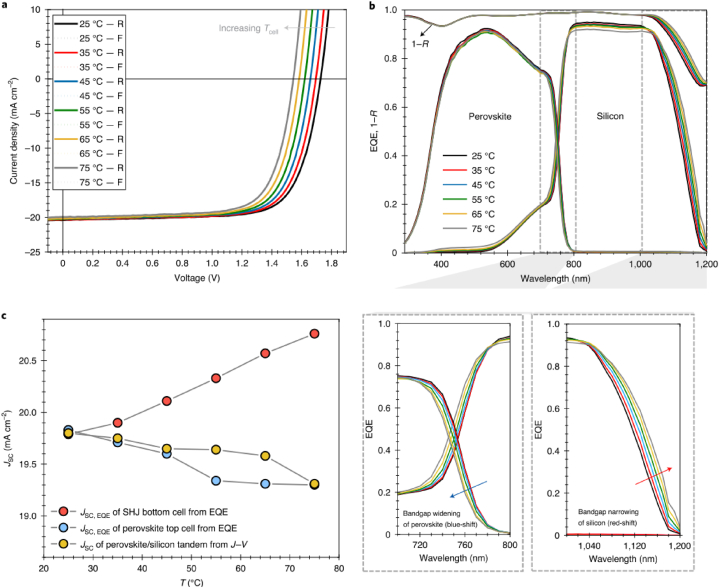
Dependence of performance of perovskite/silicon tandem cells on temperature. Panels (a) and (b) depict the temperature-dependent J–V (reverse scan denoted as R, forwards scan denoted as F) and EQE characteristics, respectively, of these devices. Panel (b) includes a magnified view of the band edge region for both sub-cells. Panel (c) presents the temperature-dependent change of EQE-measured current density (J_SC_,EQE) values for both sub-cells alongside the J–V-measured current density (J_SC_) of a tandem solar cell, indicating current limiting conditions. Reprinted with permission from Ref. [20] copyright 2020, Springer Nature. (For interpretation of the references to color in this figure legend, the reader is referred to the Web version of this article.)

Paraskeva et al. reported the outdoor performances of two perovskite active layers, one with formamidinium chloride (FACl) additive, while the other do not have any additive. As it can be seen from Fig. 4b the open circuit voltage increases during the performance enhancement at open circuit loading. The decrease in the gradient of the majority carriers’ quasi-Fermi level can be caused by the redistribution of mobile ions; this also increases the open circuit voltage. In this outdoor testing, the perovskite modules with additives demonstrated faster degradation than those without additives, implies that the benefit of the additives is not observed. The diurnal cycle outdoor testing shows that the modules efficiency is high in the morning and low in the afternoon [27] (see Fig. 5).

Aydin et al. [10,20] demonstrated that the band gap of pervoskite solar cells increases (blue shifted) with temperature, which breaks the usual trends of silicon where the band gap decreases (red shifted) with temperature. The suitable elevated temperature for outdoor testing at strong irradiance is above 25^0^C. The optimum corresponding band gap energy of the perovskite is below 1.37 eV. The report confirmed that the band gap energy of perovskite for outdoor operation at a temperature above 55^0^C is less than 1.68 eV [20].

According to the reports of the paper, the J_SC_, which dictates the energy yield of the device over a given day, is decreased by about 0.5 mA/cm^2^ in 1Sun illumination. The group used open–rack test configuration on a rooftop for outdoor testing and found that the maximum temperature of the cell under investigation to be 60°C. As a result, 75°C is assumed to be the realistic temperature for rooftop measurements at high solar irradiance. They conducted J-V scans (both in the forward scan and reverse scan) controlled laboratory environment to study its dependence on temperature by changing the temperature from 25°C to 75°C [20].

The team demonstrated that a decrease in J_SC_, a narrowing in c-Si band gap energy and a broadening in perovskite band gap energy (E_g_) are observed with increase in temperature. The team studied the properties of the device in both controlled laboratory and outdoor conditions and found that optimum outdoor performance of the tandem device corresponds to perovskite E_g_at STCs (25°C) [20]. Velilla et al. [16] studied the performance of perovskite solar cells under outdoor conditions and found that the open circuit voltage of perovskite cells exhibited a nonlinear behaviour with enhanced performance with temperature at high irradiance. They found higher short circuit current density for perovskite modules as compared to silicon modules. Liu et al. [19] fabricated monolithic perovskite/silicon solar cells from a textured silicon heterojunction solar cell that possess a stabilized PCE of 28.6 %.

**Fig. 6:**
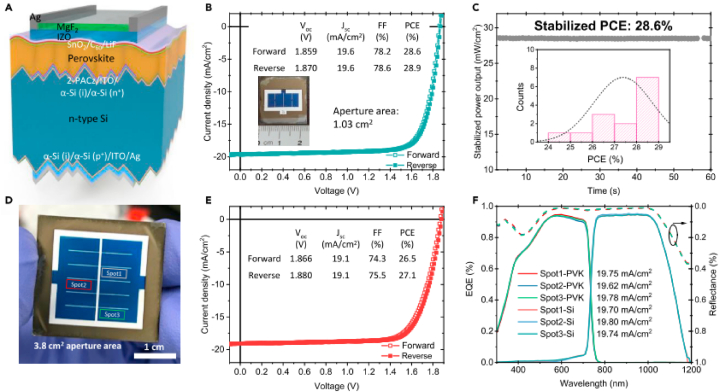
Perovskite/silicon tandem device (A) Schematic diagram of textured monolithic device (B) JV-scan of the device with an aperture mask of area 1.03 cm^2^. (C) Stability test of the device at the maximum power point (MPP) under AM 1.5G illumination. (D) Photographic image of the device with an aperture area of 3.8 cm^2^. (E) J-V scan (both in the forward scan and reverse scan) curves of the tandem device an aperture area of 3.8 cm^2^. (F) EQE spectra of the device due to three different spots.). Reprinted from Ref. [19] Copyright 2021, with permission from Elsevier. (For interpretation of the references to color in this figure legend, the reader is referred to the Web version of this article.)

**Fig. 7:**
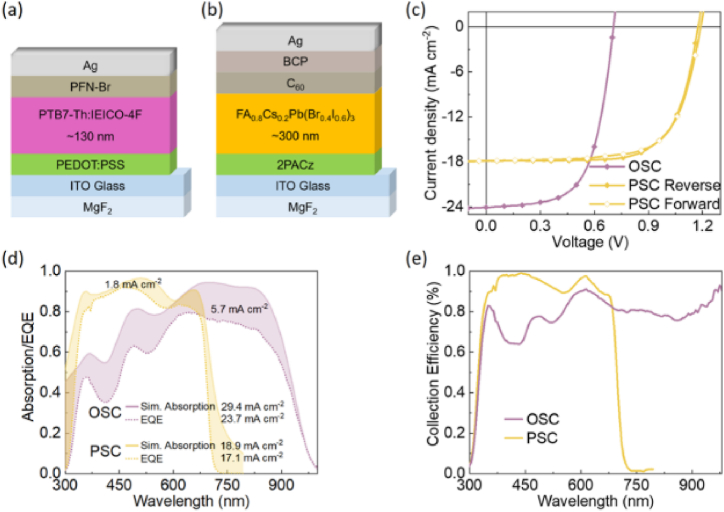
(a, b) Device architectures of organic solar cells and perovskite solar cells, respectively. (c) JV-scans of the devices indicating hysteresis effects. (d) Simulated absorption in PTB7-Th:IEICO-4F (pink) and FA_0.8_Cs_0.2_Pb(Br_0.4_I_0.6_)_3_ (yellow) (solid line) and measured (dotted line) EQE spectra of their single-junction devices. (e) Calculated collection efficiency spectra for OSC and PSC single-junction devices. Reprinted with permission from Ref. [10]. Copyright 2022, American Chemical Society. (For interpretation of the references to color in this figure legend, the reader is referred to the Web version of this article.)

In the tandem device under study, the conformal coated 2PACz layers on 20 nm ITO layers serve as the recombination junction. The PCE of the device is observed to be 28.9 % with negligible hysteresis in the JV-curve (Fig. 6 B). The stabilized PCE obtained using maximum power point (MPP) measurements at operating voltage of 1.58 V is recorded to be 28.6 %. The device with active area 1.03 cm^2^ achieved best PCE of 27.1 % and V_OC_ of 1.88V (see Fig. 7).

Aydin et al. [20] also conducted outdoor measurements for 7 days. By changing the temperature from 25 °C to 75 °C they investigate the effect of temperature on the band gap energy and then on the performance of perovskite/silicon tandems, and found that the E_g_ of c-Si narrows as the temperature increases, while E_g_ of the perovskite widens as temperature increases [20]. It means that there are unique optimum band gap energies that can best perform under realistic conditions and standard testing conditions.

In their outdoor stability study of tandem devices, Xu et al. [10] used reverse JV-scan at a scan rate of 50 mV/s where the tandem device was held at the maximum power point between the JV-scans, while the single junction device was held at open circuit voltage in between the measurements to study the outdoor operation of such devices. Encapsulated devices were mounted on a rooftop and JV-scans were performed in 10 min interval to carryout outdoor measurements. According to this report, the perovskite device PCE is 14.3 %, with a current loss of 1.8 mA cm^−2^ [10].

Pitchaiya et al. [11] conducted JV-measurements at peak irradiance of AM 1.5G to explore the PV parameters such as J_SC_, V_OC_, and FF under outdoor conditions. The study demonstrated that C-LSPSC devices shows better performance under direct sunlight as compared to under indoor condition resulted from higher FF and V_OC_. It implies that better extraction of efficiency for the generated carriers, and shows a decrement in the series resistance at the perovskite/carbon interface. The outdoor testing environment corresponds to very harsh conditions for unencapsulated devices which significantly affects the device stability. The PV parameters of the BLD and IND devices with interfacial layers are enhanced and observed to be PCE = 5.88 and 4.77 %, FF = 58.25 and 55.05 %, and V_OC_ = 0.989 and 1.01 V, respectively [11]. The report of this study demonstrates that, the BLD devices possess very high performance with PV parameters of J_SC_ = 10.31 mA/cm^2^, V_OC_ = 0.888 V, and FF = 47.95 %, and PCE = 4.39 %. According to the JV-characteristics of the STD device, the solar cell parameters without interfacial layer is observed to be lower than the solar cell parameters of that of the other two interface-engineered IND and BLD devices. According to this report BLD devices showed significant PV performance when tested even under very harsh environmental conditions such as normal drinking water, cold water, and hot water [11].

## 7 Outdoor stability of perovskite devices

The outdoor performance and long term stability of perovskite devices depends on temperature and intensity. Bastiani et al. [29] reported the long term stability (in the order of months) to demonstrate the significance of lamination on the panel on the degradation. As outdoor tests response of a peorvskite solar cell vary every day, stability study under realistic conditions is rather difficult [3]. Using additives, it is possible to improve the outdoor stability of perovskite devices, though T_80_ of 1000 h in a damp heat test could not be achieved. During outdoor testing, the devices can be kept at open circuit, fixed operating voltage maximum power point (MPP), or Maximum power point tracking (MPPT) based on the desired ISOS protocols.

Stability tests under outdoor operation demonstrate the real operation of the devices. It also gives insight for the stability of cells, modules, and tandem devices. Outdoor tests performed on nine solar farm based on graphene–perovskite panels with area 0.5 m^2^ each, and each panel consisting of 40 modules demonstrated a T_80_ as high as 5832 h [3,8].

**Fig. 8:**
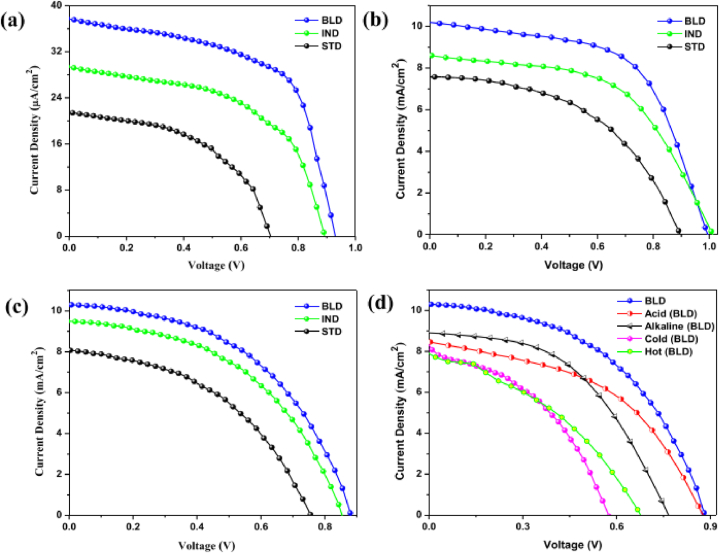
JV-scan of STD, IND, and BLD based C-LSPSC devices (a) under indoor lab testing conditions illuminated with LED of irradiance about 0.1 Sun (b) under outdoor illumination of 1 Sun at AM 1.5G (c) exposed to water soaked conditions under 1 Sun illuminations (d) JV-scan of BLD device under exposure of different harsh water conditions such as normal water, cold water, hot water, alkaline water, and acidic water. Reprinted with permission from Ref. [11]. Copyright 2022, American Chemical Society.

Paraskeva et al. studied the degradation rates, and observed that the one with additive demonstrated faster degradation under open circuit loading conditions between JV-scans. The long–term operation of both modules shows a decrease in PCE during the day, followed by recovery over the night. The modules with additive and without additive demonstrate a normalized diurnal performance degradation of 15–20 % and 10–15 % respectively, in their outdoor testing lifespan. Before this work, there was no concurrent long term outdoor testing with and without such additives (see Fig. 8).

**Fig. 9:**
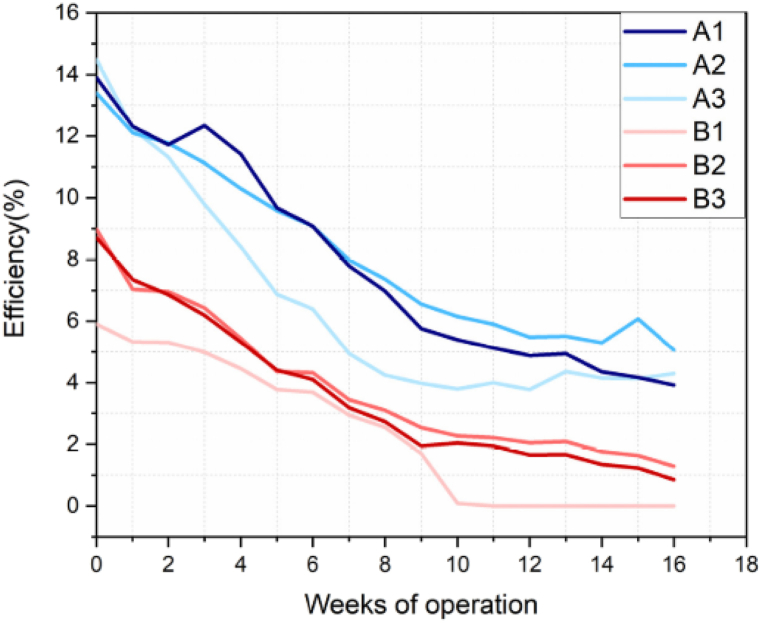
Long term operation outdoor performance test of perovskite modules without additive (module A) and with additive (module B) under reverse scan. Reprinted from [24] in accordance with Creative Commons Attribution (CC-BY) license 4.0 Copyright 2023, MDPI.

It is observed from Fig. 9 that the efficiency of all modules drops rapidly (by about 25 %) in the first 6 weeks though there is variations in decrement among the modules. Modules with additive possess open circuit's voltage between IV scans and outdoor exposure, this lead to the reduction in efficiency. During stability testing, open circuit voltage constant means that there is no change in non-radiative recombination rate, which indicates the amount of defect is not changing. The change in current density (Fig. 4a) is associated with the migration of ions, which affects the charge carrier extraction [27]. Paraskeva's group used 6 MA free planar p-i-n architecture perovskite modules, 3 without and 3 with additive labelled as A and B respectively, for this outdoor testing. They monitored both types of modules for 16 weeks (from 12 January until the May 13, 2021). The IV measurements were taken in every 10 min for alternating forward scan and reverse scan at a scan rate of 1 V/s, while the modules left at open circuit voltage (V_OC_) between the IV scans. The measurement was conducted at a maximum irradiance of 1290 W/m^2^, maximum relative humidity of 100 %, and maximum ambient and module temperatures 37.5^0^C of 54^0^C respectively [4].

The modules PCE depicted in Fig. 9 shows that, one module with additive (module B1) completely degraded at the 10th week of outdoor operation. At the initial stage of operation, all the modules show significant decrease in performance, and started to stabilize in the first 2 weeks. The result also demonstrated that the same batches and the same type of modules exhibit different performance after weeks of outdoor operation. It is observed that type A modules possess higher PCE as compared to type B modules [27].

While studying the outdoor performance of Monolithic Perovskite/Silicon tandem, Bastiani et al. [8,12] observed that the open circuit voltage maintains its original value, while the fill factor reduces. In this study, stable performance of the device for 7500 h was reported, which exhibited T_80_ of less than 200 h when exposed to 85 % RH at 65 °C [9].

**Fig. 10:**
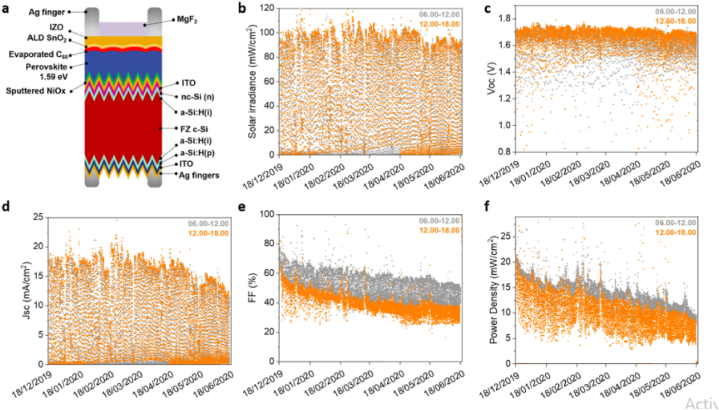
Six months outdoor testing performance of perovskite/silicon tandem devices. (a) Bifacial monolithic tandem architecture. (b) Field testing solar irradiance measured from the Nov 19, 2019 to the Jun 17, 2020. (c–f) Voc, Jsc, FF, and the power density. The gray color data is taken in the morning, while, the orange color data is taken in the afternoon. Reprinted with permission from Ref. [17]. Copyright 2021, American Chemical Society. (For interpretation of the references to color in this figure legend, the reader is referred to the Web version of this article.)

Though the open circuit voltage (Voc) of the devices remains almost the same, there are small relative loses on the short circuit current density (Jsc). Bastiani and his team reported an initial PCE of 23 % for these devices. The solar cell parameters dependence on the incident irradiance is demonstrated in the Fig. 10c-f where one sample is tracked in every 10 min during light hours, and kept at open circuit when there was no light (in between the measurements) for a period of six months. The V_OC_ depends on the intrinsic property of the absorber layer manifested in the band gap energy, while the J_SC_ show increment with the irradiance. The FF shows the continuous degradation of the device which drops from 80 % to 50 % in six months of ou.tdoor testing.

**Fig. 11:**
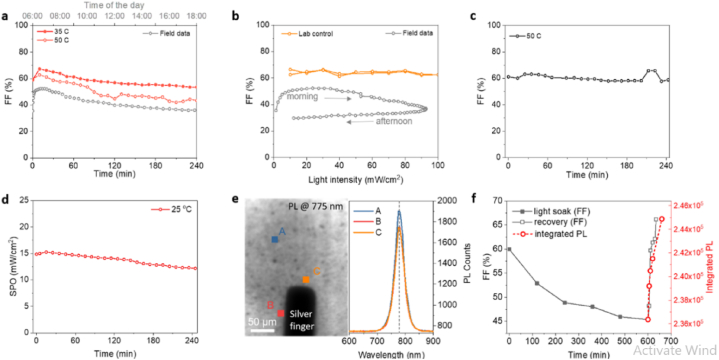
Reversible degradation of perovskite solar cells. (a)For the same level of irradiance (1SUN) at 50° C and 35° C, the FF changes compared with the outdoor testing data of the last day. (b)The FF changes with change in the light intensity in the lab as compared to the FF changes in the last day of the field test measurement data. (c) The FF affected by annealing the perovskite solar cell in the dark where there was an interruption in every 10 min until JV measurements conducted. (d) Stabilized power output of the device at 25 °C. (e) PL mapping (left) and PL spectroscopy of the perovskite device(right). (f) FF and PL recovery of light soaked devices (at 1-Sun for 10 h at open circuit condition). Reprinted with permission from Ref. [17]. Copyright 2021, American Chemical Society.

They conducted comparison of the current density as a function of voltage for outdoor and lab testing conditions. In most outdoor testing, solar cells are maintained near the maximum power point (MPP) than being in open circuit conditions [17].

Babics et al. observed that the outdoor performance of Perovskite/silicon tandem devices maintains 80 % of its PCE after one year outdoor operation [13]. According to this report, the power is stabilized in the first week, while the V_OC_ increased from 1.71 V to 1.77 V, and then become stable for 8 months, and observed to be 1.75 V after one year of operation. However, it was increased to 1.8 V when the outdoor temperature lowers. The FF stabilized immediately and then increases from 74 % to 78 % during the first week. Passivation of the devices stabilizes the power as manifested in the V_OC_.

**Fig. 12:**
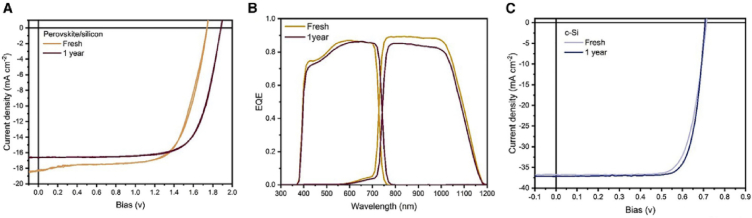
One year outdoor operation of encapsulated perovskite devices (A and B). (A) JV scan when fresh and after one year. (B) EQE when fresh and after one year filed test (C) JV scan of the reference silicon hetrojunction device when fresh and after one year. Reprinted from Ref. [13] Copyright 2023, with permission from Elsevier. (For interpretation of the references to color in this figure legend, the reader is referred to the Web version of this article.)

**Fig. 13:**
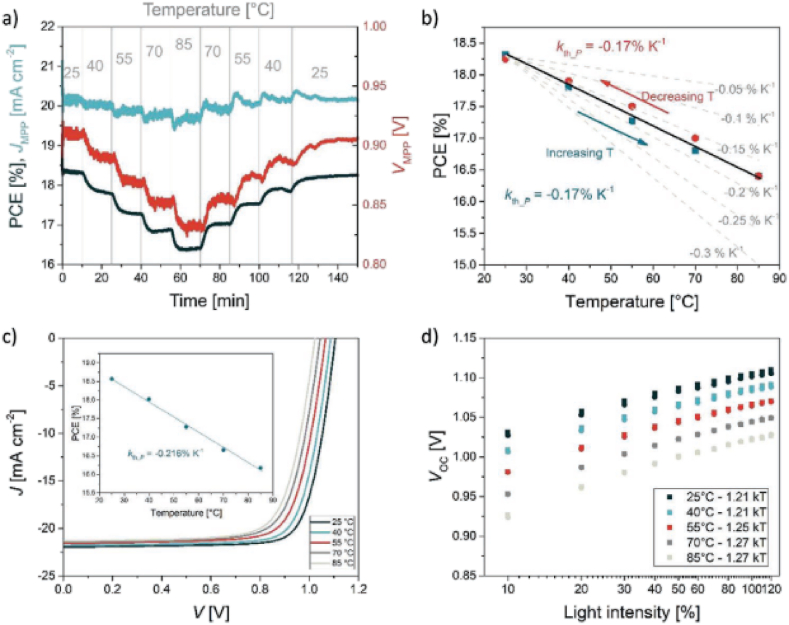
Outdoor testing of a single junction perovskite device. a) MPP track of the device at different temperatures between 25 and 85 °C. b) Variation of PCE with temperature where the points were taken from MPP data. c) JV-scan of the device at 100 mW/cm^2^ incident irradiance. d) The effect of temperature and incident light intensity on V_OC_ of perovskite device. Reprinted from Ref. [15] in accordance with the International CC-BY Creative Commons Attribution license 4.0 Copyright 2020, John Wiley and Sons.

For the first four months, the FF was sustained above 78 % and then slowly reduced to 70 % after one year of operation. The FF is the most degrading PV parameter under outdoor conditions, and hence its results favour to get stable tandem technology. After one year outdoor operation, the Jsc drops from 18.2 to 16.7 mA/cm^2^ (Fig. 12A) whereas the device retains 80 % of its initial efficiency. Moreover, the EQE decreases (Fig. 12B) which indicates the degradation of electrical contacts and decrease in the charge collection efficiency [9]. This is probably the maximum long-term outdoor stability testing reported until this review is conducted (see Fig. 13).

Jošt et al. [15] conducted the first real MPP tracking of single-junction perovskite solar cells under outdoor conditions and verified with indoor lab testing. They prepared 1 cm^2^ size perovskite devices with an average lab PCE of 18.5 % and placed on the rooftop after encapsulated. The group used planar p-i-n device with architecture glass|ITO|MeO-2PACz |perovskite|C_60_|SnO_2_|Cu which can withstand degradation as compared to n–i–p spiro-OMeTAD based devices. They conducted the outdoor measurement on a rooftop at University of Ljubljana from August to November [15].

The performance of the device decreases at elevated temperature due to the decrease in V_OC_ indicating the perovsike devices are proper for monitoring at elevated temperature [15]. Perovskite devices operating under diurnal cycles outdoor testing poses reversible degradation [30].

**Fig. 14:**
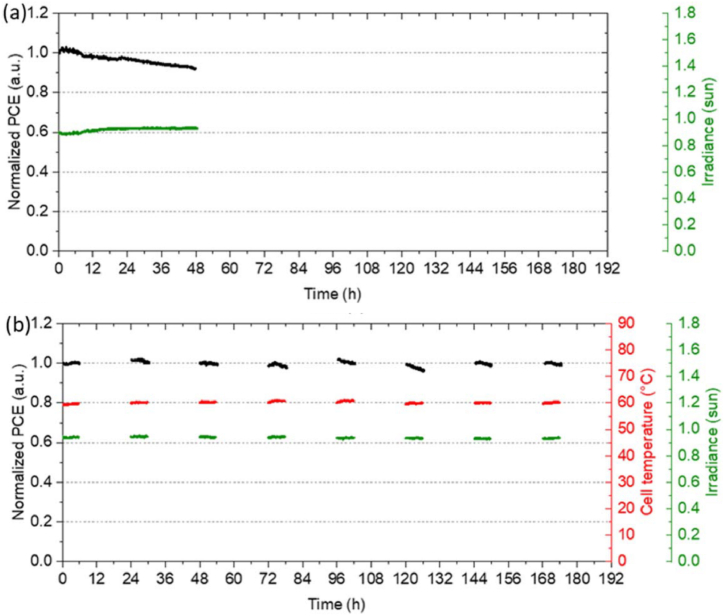
Non-encapsulated PSCs tests under outdoor stresses for eight days. The device configuration is ITO/SnO_2_/PCBM/FAMAPbIBr/Spiro-OMeTAD/Au and kept at solar irradiance of 1Sun and a temperature of 60 °C during illumination for both cases, while kept at 25 °C during the night. Reprinted from Ref. [30] under the terms of the Creative Commons Attribution 3.0 license Copyright 2020, IOP Publishing.

**Fig. 15:**
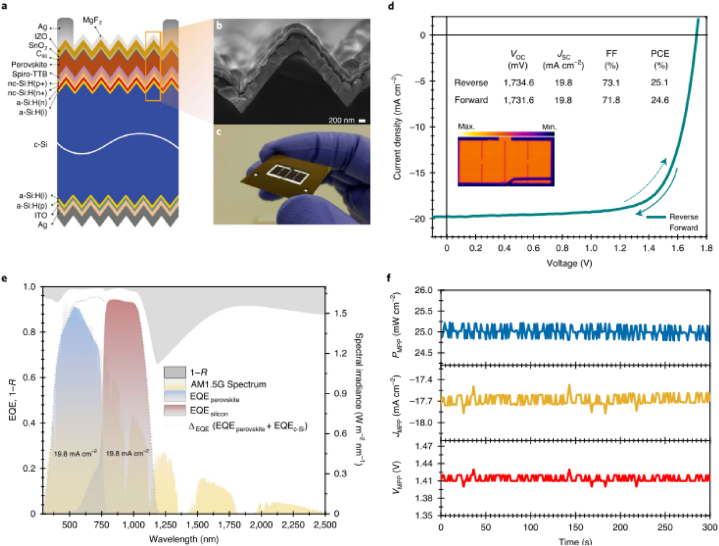
Device architecture and performance of perovskite/silicon tandem solar cells. (a) Cross-sectional morphology of the device using scanning electron microscopy (b) photographic image (c) perovskite/slicon tandem (double side textured)solar cell. (d) JV-sweep of perovskite/silicon tandem device that has a certified PCE of 25 % and band gap energy of the perovskite absorber layer 1.63 eV. (e) The EQE the corresponding device at 25 °C and AM1.5G. The integrated values of the EQE of each sub-cell give rise to the J_SC_. (f) The solar cell parameters (power, current density, voltage) all at the MPP of a perovskite/silicon tandem operating at the MPP tracking. Reprinted with permission from Ref. [20] copyright 2020, Springer Nature. (For interpretation of the references to color in this figure legend, the reader is referred to the Web version of this article.)

**Fig. 16:**
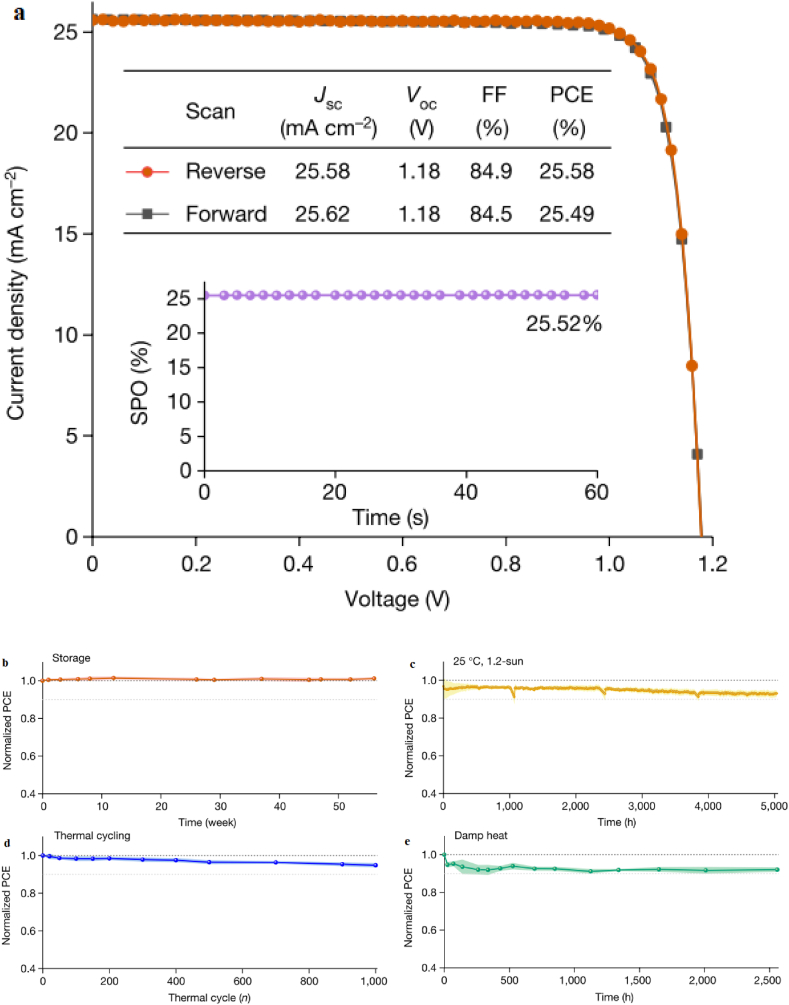
Characterization of a p-i-n (inverted) perovskite with configuration (glass/ITO/MeO-2PACz/perovskite/C_60_/SnO_2_/Ag with perovskite composition Rb_0.05_Cs_0.05_MA_0.05_FA0_0.85_Pb(I0_0.95_ Br_0.05_) _3_ (a) J-V both in the forward and reverse scan with corresponding solar power output.(b).Comparison of the solar cell parameters of the device with different devices using statistical analysis.(c) evolution of the efficiency of the unpackaged device when stored in dark (d) Continuous monitoring of the device operation under 1.2sun illumination at 25^0^C. (e) Evolution of the efficiency for packaged device under recurrent thermal recycling in the dark (f) Perosvkite devices testing under damp heat at temperature of 85^0^C and relative humidity 85 %. Reproduced with permission from Ref. [31]. Copyright 2023, Springer Nature.

Song and Aernouts [12], considered two scenario analysis to study the degradation of FA and MA containing double cation perovskite (Fig. 14). The first scenario (a) continuous degradation and the second scenario (b) there is degradation, but recovered during nights. Despite the same stressing conditions such as the same temperature and irradiance for the same time, the device becomes more instable scenario (b) as compared to (a). In both scenarios, replacing MA by Cs improves the outdoor stability (see Fig. 15, Fig. 16).

Emery et al. [14] prepared p-i-n (inverted) device with configuration ITO|2PACz|perovskite|C_60_|SnO_2_|Cu and conducted continuous outdoor monitoring on lamination-based glass−glass encapsulation for outdoor operation and commercial use (COM) devices for 10 months under maximum power point tracking on a roof-top test site in Berlin, Germany (ISOS-O3). According to this report, these devices completely retain their initial efficiency for the full duration of the outdoor test (>10 months). This is probably the second long time duration outdoor stability test until this review was made.

Jiang et al. [31] used p-i-n (inverted) perovskite devices that has a PCE of about 25.5 % and certified stabilized PCE of 24.3 % to foresee six-months outdoor tests from accelerated indoor tests. They investigated that the interface of ITO/self-assembled monolayer-based hole transport layer/perovskite significantly affects the stability of the device at 50 °C-85 °C by a factor of about 2.8 reaching over 1000 h at 85 °C and to near 8200 h at 50 °C, with a projected 20 % degradation, which is among the best to date for high-efficiency p–i–n PSCs [31].

The devices demonstrate degradations of 5 % and 8 % under thermal recycling test after 1000 thermal cycles and damp heat test after 2560 h respectively [31]. Jiang and his colleagues also investigated the device stability at high irradiance (1.2 Sun) and high temperatures varying from 25 °C to 85 °C.

As the devices operating temperature increases, the cells degrade rapidly where the T_80_ changes from 14,580 h at 25 °C to approximately 360 h at 85 °C. The J–V measurements were conducted to track device stability and the reverse-scan PCEs were used for analysis as the reverse- and forward-scan PCE evolutions are almost the same. For the samples examined in this study, the devices aged at 85 °C exhibited 40-fold faster degradation than the devices aged at 25 °C [31].

**Fig. 17:**
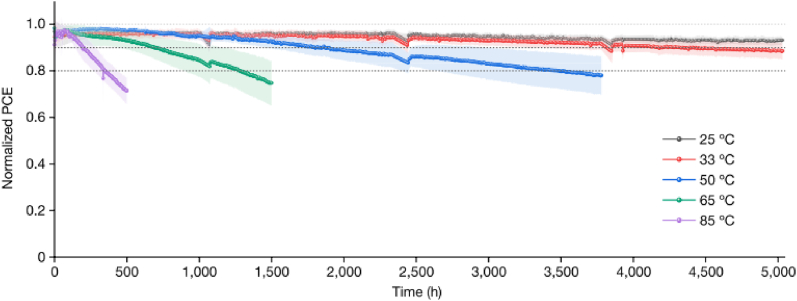
Operational stability of unencapsulated p-i-n perovskite device under high irradiance (1.2 Sun) illumination varying elevated temperatures. Reproduced with permission from Ref. [31]. Copyright 2023, Springer Nature.

**Fig. 18:**
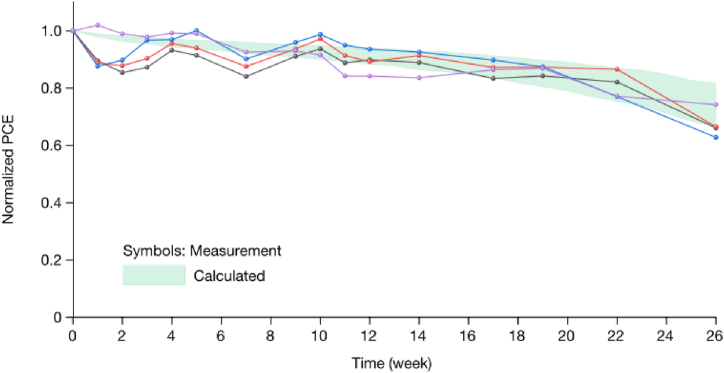
Normalized PCE of a perovskite device under outdoor testing. The device efficiency operating near the maximum power point evolves with aging. Reproduced with permission from Ref. [31]. Copyright 2023, Springer Nature.

**Fig. 19:**
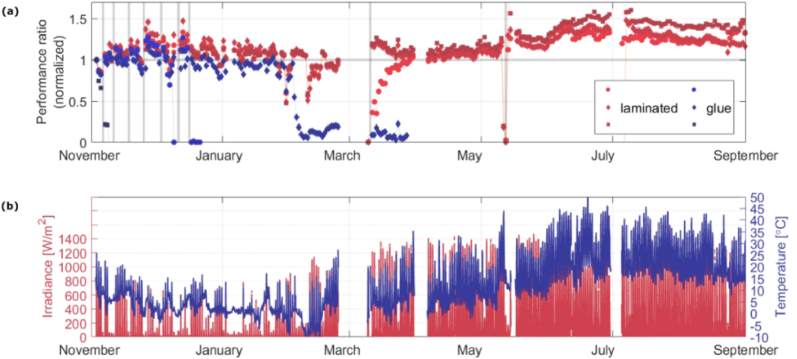
(a) Performance ratio evolution PSCs under outdoor illumination for glue (“LAB”)encapsulated and laminated (“COM”) cells. (b) Irradiance and cell temperature used for outdoor efficiency measurement. Reprinted from Ref. [14] under the terms of the Creative Commons Attribution (CC-YB) license 4.0. Copyright 2022, American Chemical Society.

The group studied the long term stability of the devices outdoor conditions by conducting periodic indoor measurements of the PCE under solar simulator to study the degradation (see Fig. 17). This helps to compare the long-term performance of the device under indoor and outdoor illumination [31]. The results of the report show that the device retains about 66–75 % its initial PCE after 26 weeks of outdoor monitoring (Fig. 18). This enables to predict the performance of the devices under outdoor operations without moisture ingress where temperature and illumination act as stressing factors. The ITO/HTL/perovskite interface region is believed to limit the stability of the device under illumination and elevated temperatures. The packaged devices were exposed to outdoor conditions for 22 weeks, and observed that 14 devices retain 90.1 % of its initial PCE. The stability was tracked using the PCE of the reverse J-V scan [31] (see Fig. 19).

Gao et al. [12] stated that the energy conversion efficiency (ECE) of optimized tandem configuration of monolithic perovskite tandem, device under outdoor testing conditions reaches to 23.3 % [20]. Emery et al. [14] demonstrated that using encapsulation it can be possible to retain the initial performance of PSCs for 3 months continuous outdoor illumination. The report stated that outdoor measurements under MPP tracking show a pronounced decrease in the performance during the 4th month outdoor testing. However, indoor control measurement shows that the device maintains 78 % of its initial efficiency after four months outdoor testing.

According to Liu et al. [19] carbazole additive encapsulated device show a PCE of 19.4 % during its first day measurement where the peak power and irradiance were 18.2 mW/cm^2^ and 937 W/cm^2^ respectively [19].

The outdoor testing of this report demonstrated that the maximum power reaches 19.3 mW/cm^2^ after 38 days of 1-Sun illumination. It means that the additive based device retains 98 % of its initial PCE. On the contrary, the PCE of the pristine device drops from 19 % to 14.6 % and its output power decreases from 17.7 mW/cm^2^ to 13 mW/cm^2^ after 40 days. In other words, the additive based device retains 93 % of its performance while the pristine device retains only 77 % of its performance after 40 days of outdoor testing. The group reported a certified PCE of 28.2 % for monolithic perovskite/silicon tandem device and found carbazole additive can significantly improve the stability of perovskite devices under outdoor exposure [19].

Wright et al.’s [24] outdoor characterization report shows that devices without carabzole treatment retain a power output of only 77 % of the initial value primarily due to the V_OC_ degradation. Babics et al. [13] reported that perovskite/silicon tandems retain 80 % of its PCE over one-year outdoor stability testing period. According to this report, the V_OC_ increased from 1.71 V to 1.77 V when passivation of NiO/perovskite interface is introduced [13].

Commonly the life time of a PV of device is defined as the time for which the device retains 80 % of its initial rated power (T_80_). It depends on multiple factors such as nature of the material and device fabrication techniques, interconnections, weather conditions, seasonal conditions, installation, shading and soiling effects, and electrical mismatch. The parameter can be obtained by long-term operation of the device under outdoor conditions at the maximum power point [32]. Fu et al. [2] reported that a printable perovskite module containing TiO_2_/ZrO_2_/carbon triple layer maintained 97.52 % of the initial efficiency after 2000 h exposure under outdoor testing conditions [2].

According to this report, there is no degradation when the cells heated up to 80^0^C, however, when heated to above 100^0^C, a steady decrease in the device efficiency is observed. The report shows that when the device is heated up to 120^0^C, the open circuit voltage (V_OC_) decreases; however, the short circuit current density (J_SC_) and the fill factor (FF) remained constant demonstrating that there is no significant degradation on the absorber layer [2]. The device performance was tracked for three months under outdoor stability testing conditions. Though the device was stored under outdoor conditions, the performance measurement was conducted in indoor using a solar simulator. The device maintained 97.52 %, 95.08 %, and 99.78 % of its initial PCE, J_SC_, and FF respectively after 2136 h of illumination, while the V_OC_ of the device increased by 2.66 % during the outdoor exposure [2].

Carlo et al. [21] manufactured nine large-area (0.5-m^2^) solar panels by connecting perovskite modules. To enhance the PCE, stability, and scalability the team used interface engineering with 2D materials. According to this report, the solar farm delivered a peak power of more than 250W that can be scaled up to the desired technology. The team reported the energy production of the solar farm over 8 months of monitoring and found a performance degradation of 20 % after 5832 h of operation [21].

Carlo and his team developed mesoporous n-i-p encapsulated perovskite device configuration to study the electrical performance under outdoor conditions. The V_OC_, I_SC_, and power of the solar PV are not affected by degradation under environmental exposure and remained stable for 500 h period showing slightly variations according to the fluctuating weather conditions. In particular, the V_OC_ went even higher than the initial values [22].

Study shows that PSCs can maintain its stability for more than 150 days under outdoor storage, 240 h of continuous operation at the maximum power output under ambient condition with relative humidity of above 80 %. The PCE drops to 50 % after 100 days of long-term outdoor stability test due to the decrease J_SC_ resulting from the perovskite composition [33].

Mohammadi et al. [26] reported that encapsulation techniques provide promising stability under full-sun continuous illumination maintaining 80 % of the device PCE after 80h measurement [26].

Pitchaiya and his colleagues [11] reported interface engineered infiltered (IND) and layer-to-layer-deposited (BLD) perovskite devices maintain 52.9 % of its initial PCE after 10 days of performance study. However, STD devices which do not have interface layer drop its initial performance down to 22 % after 10 days of stability assessment [11] (see Fig. 22).

The outdoor performance conducted for encapsulated devices demonstrated a change in PCE as function of time (Fig. 23). According to this report, the BLD device exhibit better stability with small degradation up to 31 days, while its performance drops to 81 % after 50 days of measurement. In similar context, the IND device exhibit better stability performance, maintaining above 72 % of its initial PCE tested after the same number of days. However, STD devices without interface layer exhibit very high instability and drops 53 % to its initial PCE [11].

**Fig. 20:**
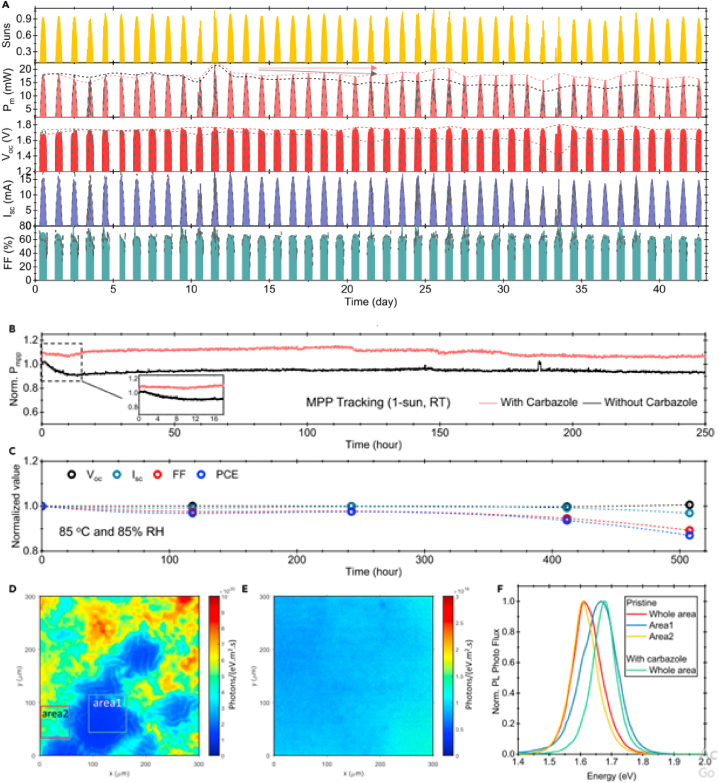
Perovskite tandem outdoor stability testing (a) performance of the device in hot climate extracted from JV-scan in the forward direction. The pristine device indicated by the dashed gray light while the carbazole treated device by color shaded areas.(b) long term measurement of the device using MPP tracking with and without additive under continuous illumination of xenon lamp.(c) evolution of the device performance under damp heat testing exposed at 85^0^C and RH of 85 %. (d & e) Post characterization of the device using PL mapping under 1-Sun illumination.(f) PL spectra of the device with and without additive. Reprinted with permission from Ref. [19] Copyright 2021, Elsevier. (For interpretation of the references to color in this figure legend, the reader is referred to the Web version of this article.)

**Fig. 21:**
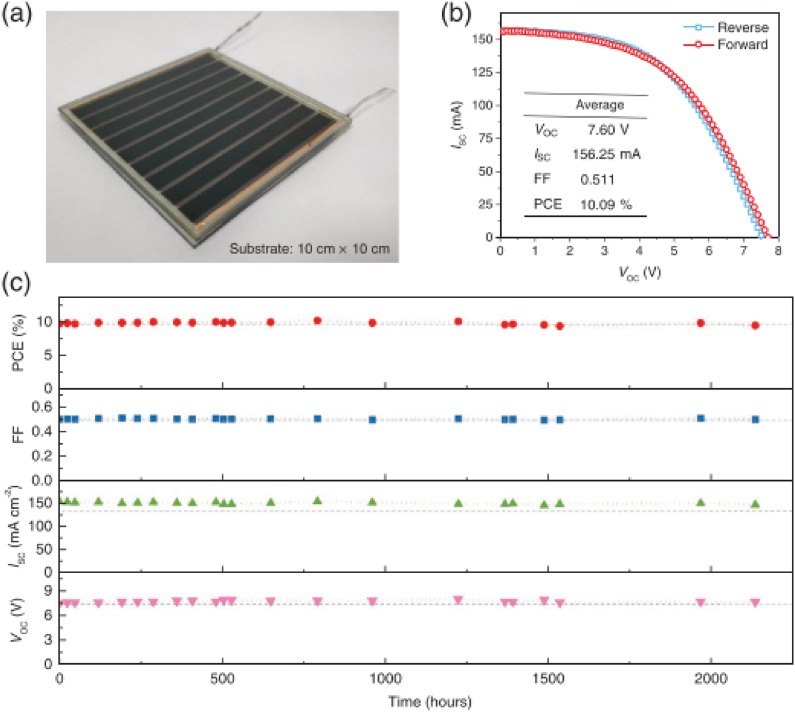
a) photographic image of a perovskite minimodule with active area 60.09 cm^2^ (b)JV-curve of the mini-module under outdoor testing (c)Evolution of the solar cell parameters under outdoor illumination. Reprinted from Ref. [2]with permission copyright 2019, John Wiley and Sons.

**Fig. 22:**
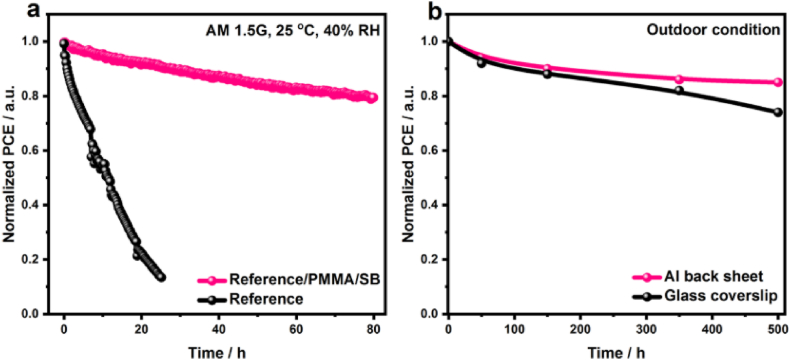
Normalized PCE indicating the stability of encapsulated and unencapsulated perovskite devices (a) under full Sun illumination and ambient condition (b) under outdoor illumination. Reprinted with permission from Ref. [26]. Copyright 2021, American Chemical Society.

**Fig. 23:**
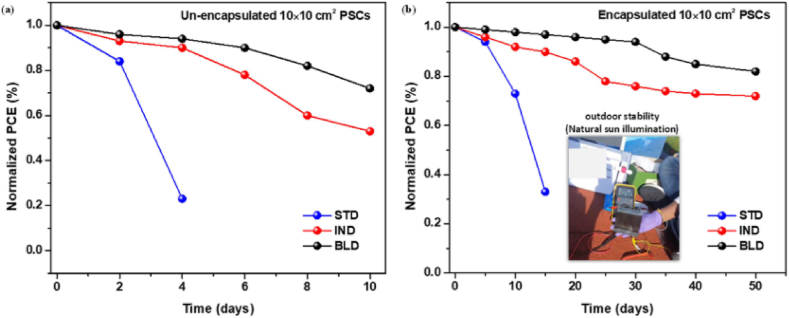
Normalized PCE as a function of time for STD, IND, and BLD based C-LSPSCs under outdoor stability testing conditions in Bergen, Norway on July 2021 to direct sunlight irradiance and measured under ambient conditions (a) for unencapsulated device and (b) for encapsulated device. Reprinted with permission from Ref. [11]. Copyright 2022, American Chemical Society.

All STD, IND, and BLD devices demonstrated strong stability during the first 6 days of continuous exposure in water soaking maintaining 85.48 %, 90.17 %, and 96 % of its initial performance respectively, after15 days of water-soaked measurements. This two-step technique prevents the decomposition of the perovskite layer resulted in an extraordinary long term stability where the device retains 85 % of its initial PCE in particular for layer-to-layer interface-engineered BLD device as compared to the other two devices.

The work reported that BLD devices completely immersed in hot water damp condition losses nearly 90 % of its initial PCE after 6 days of measurement. This could be due to melting of the encapsulate by the exposure of harsh thermal condition [11]. BLD devices demonstrated long term stability when immersed in cold water conditions as compared to hot water soaking conditions where the device retains only 20 % of its initial PCE after 9 days of measurement. BLD devices immersed in harsh alkaline water bath demonstrated excellent stability even under the exposure of corrosive environment retaining 56 % of its initial PCE after 15 days of measurements. When such devices soaked in acidic condition, it maintained 70 % of its initial PCE after 15 days measurement [11].

A summary of 2023 reports [34] on perovskite solar cells (PSCs) highlights significant advancements in research and development, with 60 published works focusing on stability. The reports indicate that inverted perovskite solar cells (IPSCs) are more favourable for commercial production due to their superior stability. The key strategies for enhanced stability include.

• Doping the hole transport layer (HTL) with materials like tryptamine (TA) for defect passivation and improved water resistance.
• Functionalizing the HTL interface with molecules like ferrocene-dithiophene-2-carboxylate (FCTC2) to reduce trap states through Pb-O binding and accelerate hole extraction, leading to high efficiency and long-term stability.
• Using self-assembled monolayers (SAMs) such as (4-(3,11- dimethoxy-7H-dibenzo[c,g]carbazol-7-yl)butyl)phosphonic acid (MeO–4PADBC) on the HTL for remarkable stability and exceptional power conversion efficiency (PCE).

These advancements in stability, PCE, and manufacturing techniques pave the way for PSC commercialization, with IPSCs showing significant promise for large-scale production in the near future.The following table shows a summary of the performance and stability of perovskite PV cells under different testing conditions**.**

**Table:** 

Device Architecture	Testing Conditions	PCE	Stability	Ref.
Encapsulated printable device with configuration **FTO/**TiO_2_/ZrO_2_/carbon/triple layer with perovskite composition of (5-AVA) _x_MA_1-x_PbI_3_	Under continuous outdoor illumination where the cells heated up to 100 °C. The outdoor exposure were kept for both daytime and night, Indoor performance measurement was conducted with solar simulator at room temperature (25 °C).	10.1 %	Maintains 97.52 % of the initial efficiency of the device after 2136 h.	[2]
n-i-p device with configuration FTO/compact TiO_2_+graphene(c-TiO_2_+G)/mesoporousTiO_2_+graphene(mTiO_2_ + G)/mixed-cation perovskite/fMoS_2_/PTAA/Au	Performance measured at 1 Sun irradiation in the reverse scan using solar simulator. The device stability tested under outdoor conditions at elevated temperature and strong irradiance, under prolonged illumination and thermal stresses.	16.4 %	Demonstrate T_80_ stability for 5832 h and T_75_ stability for 6552 h.	[8]
Perovskite/organic tandem **ITO/**SnO_2_/PEDOT:PSS/PTB7-Th:IEICO-4F/PFN-Br/Ag and ITO/2PACz/(FA_0.8_Cs_0.2_ Pb(Br_0.4_I_0.6_)_3_)/C_60_/BCP/Ag.	Performed with solar simulator under continuous illumination, and under outdoor conditions (average temperature 32 °C) for 15 days.	17.6 %	Retains 72 % of its initial PCE for 800 h, 73 % of its initial power output after 15 days of operation.	[10]
FTO/c-TiO_2_/mp-TiO_2_/perovskite (CH_3_NH_3_PbI_3-x_Cl_x_)/C	Outdoor, direct sunlight irradiance and measured under ambient conditions (12–17 °C under 70−80 % ideal humidity).	5.88 %	The champion device retains 81 % of its initial PCE for 50 days.	[11]
Perovskite/silicon tandem, Cs_0.05_ MA_0.14_ FA_0.81_Pb(Br_0.72_I_0.28_)_3_ passivated with NiOx	PCE measured under STC, subsequently tracked over one year under outdoor conditions on a fixed rack facing south with a tilt angle of 25 °C	21.4 %	Retains 80 % of its initial PCE after one year exposure	[13]
p-i-n (inverted) device with configuration ITO|2PACz|perovskite|C_60_|SnO_2_|Cu and perovskite composition Cs_0.15_FA_0.85_PbI_2.55_Br_0.45_.	Under continuous outdoor conditions, with the cells placed on an open rack (tilted 35° facing south) and connected to a maximum power point (MPP) tracking system, RH between 60 and 100 %, and indoor controlled measurements.	14.2 %	Retained its initial outdoor performance for 3 months, while 78 % of its initial indoor efficiency after 4 months	[14]
p-i-n device ITO|MeO-2PACz|perovskite|C_60_|SnO_2_|Cu with the perovskite composition Cs_0.05_(FA_0.83_MA_0.17_)Pb_1.1_(I_0.830_Br_0.17_) _3_	Under outdoor testing on a rooftop, facing south with tilt angle of 30°.	18.5 %	The devices showed excellent stability up to 85 °C	[15]
Mesoporous p-i-n configuration ITO/NiO_x_/Al_2_O_3_/perovskite/PC_60_BM/Ag with perovskite composition MAPbI_3_	Under outdoor conditions in tropical weather, facing north at fixed angle of 10°. For performance measurement, natural light used without trackers for 500 h of exposure by recording, the ambient temperature (ranging between 18 and 42 °C), irradiance (ranging between 0 and 1200 W/m^2^), and the I- V curve of the devices every minute.	15.2 %	The solar PV parameters of the PSM, Isc, Voc, and power output exhibited high stability over the 500-h period.	14]
Encapsulated bifacial perovskite/silicon tandems (p-i-n configuration)	Outdoor on a south-facing test-field structure with an inclination of 25°, matching the local latitude.	23 %	Small variations in Voc, with small losses in Jsc, losses in FF cause a drop in PCE.	[17]
Monolithic perovskite/silicon tandem p-i-n perovskite device of configuration glass/ITO/2PACz/perovskite/LiF/C_60_/BCP/Ag andabsorber layer Cs_0.05_FA_0.8_MA_0.15_Pb(I_0.75_Br_0.25_)_3_.	Conducted outdoors in a hot, sunny desert climate, with continuous operation under daily peak temperatures exceeding 45 °C and light intensity reaching 95 % of full sunlight.	28.2 %	Passivation with a carbazole additive enables the device to retain 93 % of its initial performance after 40 days.	[19]
Double-side-textured monolithic perovskite/silicon tandem solar cells (p–i–n configuration)	Measurements conducted under both controlled laboratory conditions and outdoor field conditions in a hot and sunny climate.	25.1 %	Perovskite/silicon tandem devices with narrower bandgap perovskites exhibit enhanced chemical stability.	[20]
PMMA encapsulated FTO/TiO_2_/perovskite/Spiro-OMeTAD/Au with the perovskite layer Cs_0.05_MA_0.17_FA_0.83_Pb(I_0.83_Br_0.17_)_3_.	Measurements were conducted every 24 h under damp heat conditions (85 °C, 85 % relative humidity) using a solar simulator. Outdoor conditions were characterized by average and maximum temperatures of 18 °C and 40 °C, respectively, and average and maximum humidity of 38 % and 85 %, respectively.	18.5 %	Encapsulated devices maintained 80 % of their initial PCE after 100 h, while Polymer/aluminum encapsulated devices retained nearly 90 % of their initial PCE after 500 h.	[26]
ITO/LiF/C_60_/BCP/perovskite/PTAA/ITO (p-i-n configuration) with perovskite composition Cs_0.18_FA_0.82_PbI_2.82_Br_0.18_ and one of them contains FACl additives.	Outdoor testing was conducted under conditions with a maximum ambient temperature of 37.5 °C, a maximum cell temperature of 54 °C, and relative humidity fluctuating between 11 % and 100 %.	14 % without additive and 9 % with additive.	Both pristine and additive-based devices degraded significantly within 16 weeks, retaining only 36 % and 11 % of their initial PCE, respectively. Consequently, the anticipated benefits of the additive were not realized.	[27]
ITO/PTAA/Perovskite/GABr/PCBM/BCP/(SnO_2_)/Cu with the perovskite composition FAI: MABr: MACl = 90 : 6: 9 and PbI_2_: PbBr_2_ (83 : 17).	Under AM 1.5G illumination with Xenon-lamp based solar simulator at scan rate of 20 mV/s both in the forward scan (0–1.2 V) and reverse scan (1.2–0 V) directions. The device stored in ambient conditions with relative humidity of 20%–40 % without encapsulation	17.5 % for the pristine device. 20 % when 30 nm SnO_2_ buffer layer used	The control device retains 70 % of its initial PCE, while the buffer layer assisted device maintains 90 % of its initial PCE after 600 h.	[22]
Monofacial and bifacial perovskite perovskite/c-Si tandems with different bromide-iodide ratios.	Under outdoor testing AM1.5G 1 Sun illumination, oriented at a tilt angle of 45° to south. Accelerated stability tests conducted in the dark at 85 °C, maximum power point (MPP) stability tests, and field trials.	Certified PCE >25 %.	Reducing the bromide-to-iodide ratio may enhance stability by mitigating thermal stress and charge carrier recombination.	[29]
p–i–n PSC with device configuration (glass/ITO/MeO-2PACz/Rb_0.05_Cs_0.05_MA_0.05_FA_0.85_Pb(I_0.95_Br_0.05_)/C_60_/SnO_2_/Ag).	The evolution of packaged PSCs was investigated under repeated thermal cycling between −40 °C and 85 °C in the dark, with air exposure (ISOS-T-3), and under damp heat conditions at 85 °C and 85 % relative humidity in the dark, with air exposure (ISOS-D-3). For stability tests 5–12 individual devices were used to determine average degradation.	25.5 %	Modification of the HTL enhanced stability by about 2.8 times. This translates to a projected T_80_ efficiency exceeding 1000 h at 85 °C. Demonstrate predictive capability of indoor accelerated stability tests for six-month outdoor aging.	[31]

## 8 Post characterization of perovskite devices

After outdoor testing Bastiani and his team also studied the reversibility of degradation by post characterization using photoluminescence (PL). Fig. 20e shows the PL mapping image of the device after the six-months of outdoor monitoring. The PL spectrum is centred near 778 nm (corresponds to an energy gap of 1.59 eV), which shows the presence of strong emission of the perovskite [17]. Fig. 20(d & e) shows the post characterization of the device using PL mapping under 1-sun illumination where as Fig. 20f shows the PL spectra of the device with and without additive [19]. According to Liu et al. [19] post characterization for carbazole treated device shows no change in morphology in the cross-sectional SEM images (after long term (several months) outdoor stability testing. The PL mapping report shows that pristine device demonstrated significant phase segregation [19]. The PL mapping (Fig. 20) report shows that pristine demonstrated significant phase segregation, while carbazole-treated device do not show any phase segregation. Babics et al. [13] reported the post characterization of perovskite/silicon tandems under indoor lab testing after one year outdoor testing indicates that the device a V_OC_ of 1.75 V. Babics and his colleagues the narrowing of the band gap from 1.67 to 1.63 eV [13]. Fig. 11 demonstrates the PL mapping and PL spectroscopy post characterization of a perovskite/silicon tandem device after six months outdoor testing. The device shows a narrow band emission peak at 775 nm (see Fig. 21).

## 9 Conclusion

This review summarized recent reports of perovskite solar cells and modules under outdoor conditions from 2019 to 2023 in different locations and climate conditions. Summary of outdoor electrical characterization of perovskite devices of different configurations are presented in this work. It is observed that additive engineering, passivation, testing protocols have a great impact on the outdoor performance and stability of perovskite devices. Long term outdoor testing reports shows that encapsulation and passivation with additives can significantly enhance the performance and stability of the devices. However, there are occasions which violate the stated argument. Accelerated testing of the devices under different stressors could not replace realistic outdoor measurements. Therefore, studying the performance and stability of perovskite devices under outdoor conditions in different climate conditions is very essential to realize the commercialization of the technology. Though there are very limited reports on outdoor performance and stability tests of perovskites in different climate conditions over the world, there is no certified outdoor performance and stability reports. In some regions like Africa climate conditions where there is elevated temperature and strong solar irradiance throughout the year, we did not find long-term outdoor stability reports. Thus, further outdoor testing in different climate conditions are required to scale-up the technology for commercialization at global scale.

## Data availability

There is no data collected and used for this research work, data sharing is not applicable for this paper.

## CRediT authorship contribution statement

**Getnet M. Meheretu:** Writing – review & editing, Writing – original draft, Conceptualization. **Ababay Ketema Worku:** Writing – review & editing, Supervision. **Moges T. Yihunie:** Writing – review & editing, Supervision. **Richard K. Koech:** Writing – review & editing. **Getasew A. Wubetu:** Writing – review & editing, Supervision, Conceptualization.

## Declaration of competing interest

The authors declare that they have no known competing financial interests or personal relationships that could have appeared to influence the work reported in this paper.
